# RETRACTION: Effects of Comprehensive Nursing Interventions on Wound Pain in Patients Undergoing Catheter Insertion for Peritoneal Dialysis

**DOI:** 10.1111/iwj.70448

**Published:** 2025-03-26

## Abstract

**RETRACTION:** ChenC. , WangX.‐L. , LiH. , and ZuoH. , “Effects of Comprehensive Nursing Interventions on Wound Pain in Patients Undergoing Catheter Insertion for Peritoneal Dialysis,” International Wound Journal 21, no. 4 (2024): e14795, 10.1111/iwj.14795.38572781 PMC10993332

The above article, published online on 04 April 2024, in Wiley Online Library (http://onlinelibrary.wiley.com/), has been retracted by agreement between the journal Editor in Chief, Professor Keith Harding; and John Wiley & Sons Ltd. Following an investigation by the publisher, all parties have concluded that this article was accepted solely on the basis of a compromised peer review process. The editors have therefore decided to retract the article. The authors did not respond to our notice regarding the retraction.

**RETRACTION:** C. Chen , X.‐L. Wang , H. Li , and H. Zuo , “Effects of Comprehensive Nursing Interventions on Wound Pain in Patients Undergoing Catheter Insertion for Peritoneal Dialysis,” International Wound Journal 21, no. 4 (2024): e14795, 10.1111/iwj.14795.38572781 PMC10993332

The above article, published online on 04 April 2024, in Wiley Online Library (http://onlinelibrary.wiley.com/), has been retracted by agreement between the journal Editor in Chief, Professor Keith Harding; and John Wiley & Sons Ltd. Following an investigation by the publisher, all parties have concluded that this article was accepted solely on the basis of a compromised peer review process. The editors have therefore decided to retract the article. The authors did not respond to our notice regarding the retraction.

